# The Protective Agents Used against Acrylamide Toxicity: An *In Vitro* Cell Culture Study-Based Review

**DOI:** 10.22074/cellj.2021.7286

**Published:** 2021-08-29

**Authors:** Sedat Kacar, Varol Sahinturk

**Affiliations:** Department of Histology and Embryology, Faculty of Medicine, Eskisehir Osmangazi University, Eskisehir, Turkey

**Keywords:** Acrylamide, *In Vitro*, MTT, Protective Agents, Toxicity

## Abstract

Acrylamide is a dangerous electrophile with the potency to react with many biological moieties including proteins, and nucleic acids as well as other macromolecules. Acrylamide was first only known a chemical exposed in working areas as a neurotoxicant, it was later discovered that beyond just being a neurotoxicant exposed in industrial areas, acrylamide is exposed via daily foods as well. As such, several strategies have been sought to be developed to relieve the toxic spectrum of this chemical. The utilization of a protective agent against acrylamide toxicity was one of those strategies. To date, many agents with protective potency have been investigated. Herein, we compiled these agents and their effects shown in in vitro studies. We used the search engines of Web of Knowledge and searched the keywords "acrylamide" and "protect" in the titles along with the keyword “cell” in the topics. Twenty-one directly related articles out of 35 articles were examined. Briefly, all agents used against acrylamide were reported to exhibit protective activity. In most of these reports, 5 mM concentration of acrylamide and 24-hour treatment were the employed dose and duration. Usually, the beneficial agents were pre-treated to the cells. PC12 cells were the most utilized cell line, and the mitogen-activated protein kinase (MAPK) and nuclear factor erythroid 2-related factor 2 (NRF2) pathways were the most studied pathways. This study, beside other importance, can be utilized as a guide for how the protective studies against acrylamide were done and which parameters were investigated in in vitro acrylamide studies. In conclusion, taking measures is of utmost importance to prevent or alleviate the toxicity of acrylamide, to which we are daily exposed even in our homes. Therefore, future studies should persist in focusing on mitigating acrylamide toxicity.

## Introduction

Acrylamide (C_3_ H_5_ NO) is a white, odorless, crystalline compound that was synthesized in the 1950s and used in many industrial areas such as paper industry, ore processing, cosmetics, wastewater treatment systems, construction industry and so on. Acrylamide is also exploited in laboratories to make polyacrylamide gel. It is also present in cigarette smoke. Acrylamide toxicity was first started to be investigated due to neurological disorders observed in workers, hence acrylamide was known as a neurotoxic agent (1, 2). In 1994, it was added into the group 2A substances (probably carcinogenic agents to humans) by the International Agency for Research on Cancer (3).

The acrylamide drew considerable attention in 2002 when Tareke et al. (4) discovered that acrylamide is formed in food. Upon this discovery, it was perceived that acrylamide was actually present in our daily foods and we were exposed to it without awareness. Since then the studies related to acrylamide have increased and the formation of acrylamide has been tried to be prevented or reduced. In addition, search for the substances that inhibit the effects of acrylamide is ongoing.

Regarding acrylamide-related studies, there are several* in vivo* studies examining its neurotoxic, genotoxic, reproductive, and cancerogenic effects. The first documented toxic effect of acrylamide was its neurotoxic effect, which was known for years. It was discovered in the wake of worker’s exposure to acrylamide showing signs of neurotoxicity (5) such as ataxia, numbness in hands and feet, muscle weakness, etc. Genotoxicity and mutagenicity of acrylamide stem from its potency to form adducts with hereditary material of the body, DNA. Sega et al. (6) proved that acrylamide binds germ cell DNA and delay and spoil proper DNA synthesis. The offspring of parents exposed to acrylamide, was pointed out to have higher risk of exhibiting genetic disease (7). Acrylamide was also reported to possess reproductive toxicity. In addition, acrylamide is a reproductive toxicant. It leads to multinucleated giant cells in seminiferous tubules (8), decreases the number and quality of sperm, while increasing its abnormality (9), inflicts harm to Leydig cells thus lowering testosterone levels (10), etc. In addition, acrylamide was contended to be able to engender cancer in* in vivo* studies and accepted as a probably cancerogenic substance by the International Agency for Research on Cancer (3).

Beside the* in vivo* studies, there are *in vitro* studies published about acrylamide (8, 11). In these studies, various cell lines were used (11-13). Although there are reviews examining and summarizing specifically the neurotoxicity (5), cancerogenic effect (14), and quantification of acrylamide (15), etc. , there are few papers summarizing protective agents utilized in *in vitro* studies, to date. To our knowledge, this review is unique in examining and summarizing the protective agents against acrylamide in *in vitro* studies with terms of the used doses and pathways. Researchers can resort to this review to have knowledge about *in vitro* protective agents and how they have been investigated so far against acrylamide; in this context, this review is a candidate being reference source for future studies.

In this review, we presented the substances that have the potential to protect against the harmful effects of acrylamide as shown by *in vitro* studies. We examined the studies by categorizing and sorting them according to the organ of the utilized cell line.

### Search rules

We only used the search engine of Web of Knowledge and searched the keywords "acrylamide" and "*protect*" in the titles and the keyword “cell” in the topics. By using acrylamide in the title, we wanted to find the most related articles –acrylamide toxicity studies, considering that acrylamide can be mentioned in many articles because it is used in laboratory for polyacrylamide gel and other reasons. We used "*" at both ends of "protect". "*" provide the right and left-hand truncation in Web of knowledge. Finally, to limit the search outcome to the *in vitro* studies, especially cells, we used the keyword "cell". Twenty-one directly related articles out of 35 articles were examined. All are given in detail in Tables 1, 2.

**Table 1:** The summary of *in vitro* studies, in which protective agents were used against acrylamide toxicity


Article number of references	Cell line	Cell type	Acrylamide dose	Duration	Protective agent and treatment manner	Protective agent dose and duration

1-Hong et al. (16), 2019	BRL-3A	Normal rat liver cells	2 mM, constant	24 hours	allicin, pretreatment	3.75, 7.5, 15 and 30 µM for 2 hours
2-Yildizbayrak and Erkan (17), 2019	TM3	Normal mouse Leydig cells	1, 10, 100, 1000 µM	24 hours	curcumin, one dose, constant, co-treatment	2.5 µM for 24 hours
3-Azari et al. (18), 2019	HepG2	Human hepatocarcinoma cells	IC_50_ of 200 µM	24 hours	nano-ceria, pretreatment	50,100 and 200 µM for 30 minutes
4-Pan et al. (19), 2018	PC12	Rat pheochromocytoma cells	0-10 mM	24 hours	N-acetylcysteine, co-treatment	0.6 mM for 24 hours
5-Jiang et al. (20), 2018	IEC-6	Rat small intestine cells	5 mM	24 hours	Ganoderma atrum polysaccharide	20, 40, 80 and 160 μg/ml for 20 hours
6-Esmaeelpanah et al. (21), 2018	PC12	Rat pheochromocytoma cells	4.85 mM (IC_50_)	24 hours	Epigallocatechin gallate and epicatechin gallate	20 µM for both for 24 and 48 hours
7-Albalawi et al. (22), 2017	ARPE-19	Human retinal pigment epithelium (RPE) cells	0.7 or 1 mM	24 hours	carnosic acid, pretreatment	10 µM for 24 hours
8-Li et al. (23), 2018	HepG2	Human hepatocarcinoma cells	10 mM	24 hours	blueberry anthocyanin	5, 10, 20 µg/ml for 12 hours
9-Song et al. (24), 2013	MDA-MB-231	Human mammary cells	5 mM	20 hours	cyanidin-3-glucoside	10, 25, 50, 100 µM for 4 hours
10-Mehri et al. (25), 2012	PC12	Rat pheochromocytoma cells	5 mM	24 hours	crocin pretreatment	10, 20, and 50 µM for 24 hours
11-Song et al. (26), 2017	BV2	Microglial cells	2 mM	24 hours	lipoic acid pretreatment	50 µM for 1 hour
12-He et al. (27) 2017	PC12	Rat pheochromocytoma cells	6 mM	24 hours	epigallocatechin-3-gallate pretreatment	2.5, 5, 10 and 20 µM for 24 hours
13-Chen et al. (28), 2014	Caco-2	Human colorectal adenocarcinoma cells	5 mM	48 hours	hispidin, co-treatment	5 and 10 µg/ml for 48 hours
14-Li et al. (29), 2017	PC12	Rat pheochromocytoma cells	5 mM	24 hours	silymarin, pretreatment	12, 24, 48, 96 and 192 µg/ml for 3 hours
15-Shi et al. (30), 2018	IEC-6	Rat small intestine cells	2.5 mM	24 hours	glycated and untreated casein, pretreatment	12.5, 25, 50, 100 µg/ml, for 24 and 48 hours
16-Chen et al. (31), 2013	Caco-2	Human colorectal adenocarcinoma cells	5 mM	48 hours	myricitrin, co-treatment	2.5, 5, 10 µg/ml for 48 hours
17-Rodriguez-Ramiro et al. (32), 2011	Caco-2	Human colorectal adenocarcinoma cells	5 mM	different times	cocoa polyphenolic extract, its polyphenols epicatechin and procyanidin B2, pretreatment	10 µg/ml of the extract, 10 µM of others for 20 hours
18-Rodriguez-Ramiro et al. (33), 2011	Caco-2	Human colorectal adenocarcinoma cells	5 mM	different times	hydroxytyrosol, pretreatment	5, 10, 20, 40 and 100 µM for 20 hours
19-Sumizawa and Igisu (34), 2009	SH-SY5Y	Human neuroblastoma cells	3 and 5 mM	18 hours	carboxyfullerene, pretreatment	60 µM for 1 hour
20-Zhang et al. (35), 2009	HepG2	Human hepatocarcinoma cells	5 or 10 mM	24 hours	hydroxytyrosol, pretreatment	12.5, 25 and 50 µM for 30 minutes
21-Cao et al. (36), 2008	HepG2	Human hepatocarcinoma cells	2.5 to 20 mM	24 hours	curcumin- pretreatment	2.5 μg/ml for 2 hours


**Table 2:** The results of *in vitro* experiments, in which various protective agents were used against acrylamide toxicity in various cell lines


Article number of references	Method	Acrylamide’s effect and dose	Protective agent’s effect and dose

1-Hong et al. (16), 2019	-ROS formation	↑	all ↓
	-SOD	↓	15 and 30 µM↑
	-GPx1	↓	7.5, 15 and 30 µM↑
	-DNA fragmentation	↑	7.5, 15 and 30 µM↑
	Western Blot		
	-pJNK/JNK	↑	all↓
	-pERK/ERK	↓	all↑
	-p-p38/p38	↑	all↓
2-Yıldızbayrak and Erkan (17), 2019	-Cell viability	10, 100, 1000 µM ↓	↑
	-Apoptosis and necrosis	10, 100, 1000 µM ↑	↓
	-OH^-^ production	10, 100, 1000 µM ↑	100, 1000 µM ↓
	-H_2_O_2_ production	1000 µM ↑	NS
	-MDA	10, 100, 1000 µM ↑	100, 1000 µM ↓
	-SOD	10, 100, 1000 µM ↓	NS
	-CAT	100, 1000 µM ↓	100, 1000 µM ↑
	-GPx1	10, 100, 1000 µM ↓	all doses ↑
	Western Blot		
	-pJNK/JNK	10, 100, 1000 µM ↑	10, 100, 1000 µM↓
	-pERK/ERK	10, 100, 1000 µM ↑	100, 1000 µM↓
	-p-p38/p38	10, 100, 1000 µM ↑	1000 µM↓
3-Azari et al. (18), 2019	-Cell viability	↓	200 µM ↑
	-ROS formation	↑	all ↓
	-MDA	↑	100 and 200 µM ↓
	-GSH	↓	100 and 200 µM ↑
	-Protein carbonyl	↑	100 and 200 µM ↓
4-Pan et al. (19), 2018	-Cell viability	↓	↑
	-ROS formation	↑	↓
	-MDA	↑	↓
	-TNF-α	↑	↓
	-IL-6	NS	NS
	Western Blot		
	-Nrf-2	↑	↑
	-NFκBp65	NS↑	↓
	**Note:** *non-significant increase		
5-Jiang et al. (20), 2018	-LDH	↑	all doses ↓
	-ROS	4-hour AA↑	all doses ↓
	- Annexin V/PI (apoptosis)	24-hour AA ↑	all doses ↓
	-Disrupted MMP	4-hour AA ↑	all doses ↓
	-SOD	16-hour AA ↓	40 µg/mL doses ↑
	-GPx1	16-hour AA ↓	all doses ↑
	-MDA	16-hour AA ↑	all doses ↓
	Western Blot (4-hour AA)		
	-Bax	↑	all doses ↓
	-Bcl-2	↓	all doses ↑
	-Casp3	↑	all doses ↓
	-Cleaved casp3	↑	all doses ↓
	-Casp9	↑	all doses ↓
	-Cleaved casp9	↑	all doses ↓
	-Cyt c	↑	all doses ↓
6-Esmaeelpanah et al. (21), 2018	-Cell viability	↓	both agents ↑
	-GSH	↓	both agents ↑
	-MDA	↑	10 and 20 µM for ECG
			↓, 20 µM for EGCG↓
7-Albalawi et al. (22), 2017	-Cell viability	↓	↑
	-TUNEL (apoptotic cells)	↑	↓
	-ROS	↑	↓
	-SOD	↓	↑
	-MDA	↑	↓
	-GSH	↓	↑
	-CAT	↓	↑
	Real-time PCR		
	-Casp 3	↑	↓
	-Casp 9	↑	↓
	-Nrf2	↓	↑
	-GPx1	↓	↑
	-SOD1	↓	↑
	-CAT	↓	↑
	-NQO1	0.7 M NS, 1 mM↓	↑
	-GCLM	NS	↑
	Western Blot		
	-Nrf2	↓	↑
8-Li et al. (23), 2018	-Cell viability	↓	all AE doses ↑
	-SOD	↓	all AE doses ↑
	-CAT	↓	all AE doses ↑
	-MDA	↑	all AE doses ↓
9-Song et al. (24), 2013	-Cell viability	↓	↑
	-LDH	↑	↓
	-ROS	↑	25, 50, 100 µM ↓
	-GSH	↓	10, 25, 50 µM ↑
	-GPx1	↑	all C3G doses ↓
	-GST	↑	all C3G doses ↓
	Western Blot		
	-GPx1	↓	all C3G doses ↑
	-GSTP1	↓	all C3G doses ↑
	- λ-GCS	↓	all C3G doses ↑
	-CYP2E1	↑	50 and 100 µM ↓
10-Mehri et al. (25), 2012	-Cell viability	↓	all CRO doses ↑
	-DNA fragmentation	↑	20 and 50 µM ↓
	-Annexin V/PI (apoptosis)	↑	all CRO doses ↓
	-Annexin V/PI (necrosis)	↑	20 and 50 µM ↓
	- ROS	↑	all CRO doses ↓
	Western Blot		
	-Bax	↑	↓
	-Bcl-2	↓	NS (Western bands
			with similar with acrylamide group)
	-Bax/Bcl-2	↑	↓
11-Song et al. (26), 2017	-Cell viability	↓	↑
	AO/EB	NQ	NQ
	-PGE2	↑	↓
	-TNF-α	↑	↓
	-IL-1β	↑	↓
	-NO	↑	↓
	-GSH	↓	↑
	-H2O2	↑	↓
	Western Blot	↑	↓*
	-p-ERK/ERK	↑	↓*
	-pp38/p38	↑	↓*
	-p-JNK/JNK	↑	↓*
	-p-IκB/IκB	↑	↓*
	-NFκB-nuclear	↑	↓*
	-Bax	↓	↑*
	-Bcl-2	↑	↓*
	-Cyt-c	↑	↓*
	-Cleaved-Casp3	↓	↑*
	-pAKT	↑	↓*
	-p-GSK-3β	↑	↓
	-Bax/Bcl-2	↑	↓*
	-MPO		
	*note: The change was inferred from the density of Western bands based on observation because most western bands had not been quantified.		
12-He et al. (27), 2017	-Cell viability	↓	-5, 10 and 20 µM ↑
	-Acetylcholinesterase activity	↓	-5 and 10 µM↑
	-Annexin V/PI	↑	-5 and 10 µM↓
	-MMP	↓	-5 and 10 µM↑
	- Intracellular Ca^+^^2^	↑	-5 and 10 µM↓
	-Cyt c	↑	-5 and 10 µM↓
	-Casp3	↑	-5 and 10 µM↓
	-ROS	↑	-5 and 10 µM↓
	-SOD	↓	-5 and 10 µM↑
	-MDA	↑	-5 and 10 µM↓
	-GSH	↓	-5 and 10 µM↑
	Real-Time PCR		
	-Bax	↑	↓
	-Bcl-2	↓	NS
	-Bax/Bcl-2	↑	↓ (only 5 µM EGCG was used)
13-Chen et al. (28), 2014	-Cell viability	↓	all HP doses ↑
	-ROS	↑	all HP doses ↓
	-MMP (rhodamine123)	↓	all HP doses ↑
	-GSH (NDA)	↓	all HP doses ↑
14-Li et al. (29), 2017	-Cell viability	↓	-↑ 48 µg/ml for 1.5 and 3 hours SM –pre- treatments; 96 and 192 µg/ml ↑ for 1.5, 3 and 6 hours SM pre-treatments
	-ROS	↑	-48, 96 and 192 µg/ml↓
	-MDA	↑	
	-GSH	↓	-all 5 SM doses↓
	Real-Time PCR		-48, 96 and 192 µg/ml↓
	-Total Nrf-2	NA	-24, 48, 96, 192 µg/ml↑
	-Gpx1	NA	-48, 96 and 192 µg/ml↓
	- GCLC	NA	
	-GCLM	NA	-24, 48, 96, 192 µg/ml↑
	Western Blot		
	-Total Nrf-2	NA	-all 5 SM doses↓
	-Nuclear Nrf2	↑	
	-Cytoplasmic Nrf2	↓	-96 and 192 µg/ml ↑
	-Gpx1,	NA	-48, 96 and 192 µg/ml ↑
	-GCLC	NA	
	-GCLM	NA	-all 5 SM doses↓
			-48, 96 and 192 µg/ml↑
			-48, 96 and 192 µg/ml↑
			-all 5 SM doses↓
15-Shi et al. (30), 2018	-Cell viability,	↓	-all CN/GCN doses↑
	-LDH	↑	-all CN/GCN doses ↓
	-TEER	↓	-↑25 µg/ml is the best
			for CN/GCN.
	-Paracellular permeability (destruction of TJs)	↑	-↓48 hours and 25 µg/ml are the best for CN/GCN.
	Real-Time PCR		
	-ZO1, Claudin 1 and 3	↓	-CN/GCN digests for 24 /48 hours ↑
	-ZO2, Occludin		-CN digest for 24 /48 hours ↑. GCN digest for 24 hours ↑, for 48 hours NS.
	-Claudin 4	↓	-CN/GCN digests for 24/ 48 hours NS
	Western Blot		
	-ZO1, Occludin and Claudin 1	↓	-CN/GCN for 24/48 hours↑
16-Chen et al. (31), 2013	Cell viability	↓	5 and 10 µg/mL↑
	ROS	↑	5 and 10 µg/mL↓
17-Rodriguez-Ramiro et al. (32), 2011	-Cell viability	↓	EC↑<PB2↑=CPE↑
	-ROS	↑	EC↓<PB2↓=CPE↓
	-GSH	↓	EC↑<PB2↑=CPE↑
	-Casp 3	↑	EC↓<PB2↓=CPE↓
	Western Blot		
	-λ-GCS	↓	EC↑<PB2↑=CPE↑
	-λ-GST	↓	EC(NS)< PB2↑=CPE↑
	-pERK/ERK	↑	EC(NS) <PB2↓=CPE↓ EC(NS) <PB2↓=CPE↓
	-pJNK/JNK	↑	
18-Rodriguez-Ramiro et al. (33), 2011	-Cell viability	↓	all HT doses ↑
	-Caspase 3 activity	↑	10, 20, 40 µM HT doses ↓
	-GSH (fluorometric)	↓	all HT doses NS
	-ROS	↑	all HT doses↓
	-GPx1	↑	10, 20, 40 µM HT doses ↓
	-GR activity	↑	all HT doses↓
	-pJNK/JNK	↑	all HT doses↓
19-Sumizawa and Igisu (34), 2009	-Cell viability	↓	10, 30, 60 and 100 µM CF doses ↑
	-Caspase 3	↑	↓
	-SubG1 cell population (cell -cycle analysis)	↑	↓
	-Acrylamide uptake	NA	60 and 120 µM CF doses ↑
	-GSH	↓	↑
20-Zhang et al. (35), 2009	-Cell viability	↓	all HT doses ↑
	-DNA tail length (Comet assay) -	↑	all HT doses ↓
	ROS	↑	all HT doses ↓
	-DNA damage (8-OHdG)	↑	all HT doses ↓
	-GSH	↓	25 and 50 µM↑
21- Cao et al. (36), 2008	-Cell viability	↑	↓
	-ROS	↑	↓
	-DNA tail length (Comet assay)	↑	↓
	-Micronucleus frequency	↑	↓


"↑", "↓" and "NS" indicate a significant increase, a significant decrease and non-significant change, respectively, when compared to control group in the column titled acrylamide’s effect and dose, and when compared to the acrylamide group in the column titled protective agent’s effect and dose. The first arrow direction (arrows in left-hand-side) shows the significant difference of acrylamide-induced cells when compared to control, the second arrow direction (arrows in right-hand-side) shows a significant difference of protective agent cells when compared to acrylamide-treated cells. In the Table and manuscript, only the data of the reviewed article that is related to the effect of the protective agents, and the only experiments that are related to *in vitro *cell line are used and presented. AA; Acrylamide, AE; Anthocyanin extract, AO/EB; Acridine orange/ Ethidium bromide, Bax; Bcl-2-associated X protein, C3G; Cyanidin-3-glucoside, Casp; Caspase, CAT; Catalase, CF; Carboxyfullerene, CN; Caseinate digest, CPE; Cocoa polyphenolic extract, CRO; Crocin, CYP2E1; Cytochrome P450 2E1, Cyt c; Cytochrome c, EC; Epicatechin, ECG; Epicatechin gallate, EGCG; Epigallocatechin gallate, ERK; Extracellular signal-regulated kinase, λ-GCS; Gamma-glutamylcysteine synthetase, GCLC; Glutamate-cysteine ligase catalytic subunit, GCLM; Glutamate-cysteine ligase modifier subunit, GCN; Glycated caseinate digest, GPx1; Glutathione peroxidase 1, GR; Glutathione reductase, GSH; Glutathione, GSK-3β; Glycogen synthase kinase 3 beta, GST; Glutathione S-transferase, GSTP1; Glutathione S-transferase P, H2O2; Hydrogen peroxide, HT; Hydroxytyrosol, IL-1β; Interleukin 1β, IL-6; Interleukin 6, JNK; c-Jun N-terminal kinase, LDH; Lactate dehydrogenase, MDA; Malondialdehyde, MMP; Mitochondrial membrane potential, MPO; Myeloperoxidase, NA; Not applicable, there is no given knowledge about it, NDA; Naphthalene-2, 3-dicarboxaldehyde, NFκB; Nuclear factor kappa B, NO; Nitric oxide, NQ; Not quantified data, NQO1; NAD(P)H quinone dehydrogenase 1, Nrf-2; Nuclear factor erythroid 2-related factor, PB2; Procyanidin B2, PGE2; Prostaglandin E2, PI; Propidium iodide, ROS; Reactive oxygen species, SM; Silymarin, SOD; Superoxide dismutase, TEER; Transepithelial/transendothelial electrical resistance, TJ; Tight junction, and TNF-α; Tumor necrosis factor α.

### The cell lines and antioxidants used for preventing acrylamide toxicity

### Liver cells

BRL-3A cells are normal, adherent cells derived from the liver of 5 week-old buffalo rat, obtained from Hayden Coon from Carnegie Institution, Baltimore. The cells proliferate in monolayer culture with an epithelial and parenchyma-like manner. When the cells grow, they seem virtually as a hepatic thin section (37). HepG2 cells are very commonly utilized cells. They are both employed in cancer studies as a hepatocarcinoma cancer model and in molecular and toxicity studies, to reveal liver metabolism and response to several chemicals or drugs. These cells are epithelial, adherent cells, obtained from a 15-year old male patient with hepatocellular carcinoma.

### Allicin

Allicin is the chemical component of *Allium sativum*, that is garlic. Allicin demonstrates a myriad of protective activities, including, neuroprotective, antiviral, antioxidative, anti-inflammatory and free-radical scavenging activities (38, 16). Hong et al. (16) searched the protective effect of allicin on BRL-3A rat liver cells. Having treated the cells with 3.75, 7.5, 15 and 30 µM of allicin for 2 hours, they applied 2 mM of acrylamide to the cells for 24 hours. As a result, they found that allicin conspicuously reduced acrylamide-induced oxidative and DNA damage via enhancing antioxidant levels and decreasing reactive oxygen species (ROS). The group (or team) also contended allicin to exert its effect through mitogen-activated protein kinase (MAPK) signaling pathway.

### Cerium oxide

Cerium oxide is a scarce metal oxide type of the lanthanides. Its nanoparticles, which are called nano-ceria, exhibit a powerful and reusable ROS scavenging ability (18, 39). Azari et al. (18) used HepG2 liver cancer cells to gauge the protective effect of cerium oxide nanoparticles. They treated the cells with 50, 100 and 200 µM of nanoparticles of cerium oxide for 30 minutes. Afterward, they treated the cells with 200 µM of acrylamide [as the half maximal inhibitory concentration (IC_50_)] of acrylamide according to the article) for 24 hours. Cerium oxide nanoparticles showed a dose-dependent protective effect against acrylamide through increasing antioxidant levels and decreasing ROS.

### Anthocyanins of blueberry extract

Blueberry was reported to offer therapeutic effects on various diseases. Of polyphenol content of blueberry, anthocyanins are the commonly existed bioactive compounds (40). In a study using HepG2 cells, Li et al. (23) sought to explore whether the anthocyanins of blueberry extract defend the cells against acrylamide toxicity. Here, 5, 10, 20 µg/ml of anthocyanins were given to the cells for 12 hours. Thereafter, the cells were treated with 10 mM of acrylamide for 24 hours. At the end of the experiments, anthocyanins were documented to maintain the cells more viable than those treated with acrylamide, by increasing antioxidant levels and decreasing oxidant level.

### Hydroxytyrosol

Hydroxytyrosol, a phenolic phytochemical component, is present in olive oil and leaf. Zhang et al. (35) used this compound to inhibit acrylamide toxicity in HepG2 cells. Concisely, 12.5, 25 and 50 µM of hydroxytyrosol were administered to the cells for 30 minutes, followed by 5 or 10 mM of acrylamide for 24 hours. The study reported that hydroxytyrosol dose-dependently alleviated the cytotoxicity, ROS amount, DNA insult and glutathione (GSH) loss engendered by acrylamide. The authors suggested hydroxytyrosol to possess high protective capability against acrylamide-induced changes.

### Curcumin

Curcumin naturally exists in the rhizome of the plant *Curcuma longa*, and possesses strong antioxidant properties. Curcumin was used to suppress acrylamide toxicity in HepG2 cells in the study of Cao et al. (36). Briefly, the cells were treated with 2.5 μg/ml of curcumin. Then, various acrylamide doses ranging from 2.5 to 20 mM were given to the cells. The findings of the study displayed that curcumin prominently lowered acrylamide-induced DNA fragmentation, micronuclei formation, ROS generation and cytotoxic effect in HepG2 cells.

### Testicular cells

TM3 cells are adherent, epithelial Leydig cells obtained from the testis of *Mus musculus*. These cells do not respond by growing to either luteinizing hormone (LH) or follicle-stimulating hormone (FSH), but exhibit an increase in cAMP and a change in the metabolism of cholesterol in the availability of LH (41).

### Curcumin

Yildizbayrak and Erkan (17) questioned the protective effect of 2.5 µM of curcumin against acrylamide’s toxicity by utilizing TM3 Leydig cells. They co-treated the cells with constant 2.5 µM of curcumin dose and varying acrylamide doses (1, 10, 100 and 1000 µM) for 24 hours. They reported curcumin to ameliorate acrylamide-induced detrimental effects. Furthermore, the expression of acrylamide-upregulated MAPKs was lowered by curcumin.

### Neuron-like cells and neuroglia cells

Rat pheochromocytoma-derived cell line (PC12) is utilized as a neuron-like cell line and considered a suitable model for neuronal differentiation in the experiments. These cells were reported to respond nerve growth factor (NGF) protein with long, branching neuronal-like prolongations and synthesize/store catecholamines (42). These cells have been widely used for neuron-related studies. This cell line has also been commonly used while searching protective substances against acrylamide in *in vitro* studies.

### N-acetyl cysteine

N-acetylcysteine (NAC) is a commonly utilized drug with relatively high therapeutic impacts to counteract the inflammatory process and oxidative stress. It is also the precursor of non-enzymatic antioxidant GSH (28). Pan et al. (19) investigated the effects of NAC on acrylamide-treated PC12 rat pheochromocytoma cells. NAC at a dose of 0.6 mM was given to the cells that were co-treated with different acrylamide concentrations of 0- 10 mM for 24 hours. NAC treatment alleviated the increased malondialdehyde (MDA), ROS and tumor necrotizing factor-alpha (TNF-α) levels, and activated the nuclear factor erythroid 2-related factor 2 (Nrf-2) pathway while inhibiting Nuclear Factor kappa B(NF-κB) pathway.

### Catechins

Epicatechin (EC), epicatechin-3-gallate (ECG), epigallocatechin (EGC) and epigallocatechin-3-gallate (EGCG), which are the main catechins of green tea, constitute 6.4, 13.6, 19% and 59% of it, respectively. He et al. (27) used a polyphenol in green tea, EGCG against acrylamide toxicity in PC12 cells. The cells were initially pretreated with different EGCG concentrations (2.5, 5, 10 and 20 µM) for 24 hours. Later, the cells were treated with 6 mM of acrylamide for 24 hours. As a result, EGCG maintained the cells more viable and prevented apoptosis, which was shown by Annexin V, cytochrome c and caspase 3 and Bcl/Bax ratio analyses. Moreover, it lowered ROS and MDA and augmented antioxidant levels such as GSH and superoxide dismutase (SOD).

Esmaeelpanah et al. (21) utilized the catechins of green tea, EGCG and EGC to protect against acrylamide toxicity in PC12 cells. They treated the cells with 2.5, 5, 10, 20, 30 and 40 µM of EGCG and EGC for 24 and 48 hours, followed by 4.85 mM of acrylamide treatment for 24 hours. According to the cell viability assay, all treated doses displayed a higher viability percentage than acrylamide-treated cells; however, 20 µM of both agents was the optimum dose, at which maximum cell viability was detected.

### Crocin

Crocin is the principal constituent of *Crocus sativus* L., also named Saffron. It is a biologically beneficial substance with reported antioxidant activities* in vivo* and *in vitro* (25, 43). Mehri et al. (25) treated the PC12 cells with various concentrations of crocin (10, 20, and 50 µM) for 24 hours. After that, 5 mM of acrylamide was given to PC12 cells for 24 hours. With crocin pre-treatments before acrylamide administration, the amount of DNA fragmentation and ROS generation decreased, while the cell viabilities increased. In the same study, crocin was shown to decrease acrylamide-induced apoptosis by diminishing the Annexin V levels and Bax/Bcl-2 ratio.

### Lipoic acid

Lipoic acid is an organic substance with sulfur and an organosulfur compound, found in foods and can be synthesized by many animal and plant species. Song et al. (26) utilized lipoic acid to prevent acrylamide toxicity in BV2 microglial cells. The cells were initially pretreated with 50 µM of lipoic acid for 1 hour. Then, the cells were treated with 2 mM of acrylamide for 24 hours. Lipoic acid brought the mitochondrial potential and H_2_ O_2_ levels to control levels and increased the depleted GSH levels to some extent, when compared with the acrylamide-treated group. Moreover, the increased myeloperoxidase (MPO), nitric oxide (NO), TNF-α and interleukin-1 beta (IL-1β) levels decreased upon lipoic acid pretreatment while increasing the brain-derived neurotrophic factor

### Silymarin

Silymarin is an extract gained from milk thistle with many pharmacologically beneficial activities. Li et al. (29) researched the possible protective effect of this extract on PC12 cells following acrylamide administration. Briefly, they treated the cells with the silymarin doses of 12, 24, 48, 96 and 192 µg/ml for 3 hours, which was followed by 5 mM of acrylamide treatment for 24 hours. The increases in ROS and MDA levels and the decrease in GSH levels after acrylamide treatment were reversed by silymarin treatments, especially at the doses of 96 and 192 µg/ml. Furthermore, the expressions of Nrf2 and its downstream target antioxidant enzymes including glutathione peroxidase (GSH-Px), glutamate-cysteine ligase catalytic and glutamate-cysteine ligase modifier subunits were up-regulated.

### Carboxyfullerene

Carboxyfullerene is a derivative of trimalonic acid fullerene, which is readily soluble in water, and and it was reported to have antioxidant activities by scavenging ROS (44). Sumizawa and Igisu (34) utilized neuroblastoma cells to explore the therapeutic effect of carboxyfullerene. First, the most suitable dose of carboxyfullerene was determined, then the SH-SY5Y cells were incubated with 60 µM carboxyfullerene for 1 hour. After that, different acrylamide concentrations were given to the cells for 18 hours. Lactate dehydrogenase (LDH) leakage was diminished, the cell viability was enhanced, caspase 3 activity was lowered and cellular GSH content was maintained by carboxyfullerene.

### Intestinal cells

IEC-6 cells are adherent, epithelial small intestinal cells, obtained from intestines of germ-free adult *Rattus norvegicus* in the 1970s, which are reported to be derived from the crypt cells of the intestine and maintain properties of small intestinal epithelial cells (45). Caco-2 cells are adherent, epithelial cancer cells, obtained from the colon of a 72-year old male patient with colorectal adenocarcinoma and established by Jorgen Fogh in Memorial Sloan-Kettering Cancer Center, New York in 1974 (46).

### Ganoderma atrum polysaccharide

*Ganoderma atrum* is a black-colored fungus, with several documented biological activities (47, 48). In a study done by Jiang et al. (20), IEC-6 epithelial small intestinal cells were used to study the impacts of *G. atrum* polysaccharide against acrylamide-evoked oxidative injury. In the study, first, the cells were incubated with varying concentrations (20, 40, 80 and 160 μg/ml) of *G. atrum* polysaccharide for 20 hours, followed by 5 mM of acrylamide treatment. The polysaccharide counteracted the acrylamide toxicity by increasing antioxidant (SOD, GSH-Px) and anti-apoptotic (Bcl-2) protein levels and decreasing oxidant (MDA) and pro-apoptotic (Bax, caspases, etc.) protein levels.

### Hispidin

Hispidin is one of the bioactive components of the edible fungus *Phellinus linteus* belonging to the genus *Phellinus*. Chen et al. (28) attempted to disclose if hispidin has a protective effect against acrylamide-induced toxicity in Caco-2 cells. To evaluate the protective effect of hispidin, either 5 or 10 µg/ml hispidin and 5 mM of acrylamide were given to the cells for 48 hours. As a result, hispidin held the cells at a certain viability level and alleviated the ROS surge, GSH depletion and disrupted mitochondrial membrane potential induced by acrylamide.

### Caseinate

In a study carried out by Shi et al. (30), the glycated caseinate and untreated caseinate, which are two tryptic digests, were assessed if they counteract intestinal barrier deformation induced by acrylamide in the IEC-6 cells. In the study, acrylamide doses of 1.25-10 mM led to a reduction in cell viability and an increase in LDH release. In the study, acrylamide was also reported to lead to intestinal barriers abnormality by decreasing trans-epithelial electrical resistance and increasing epithelial permeability. The caseinate administration together with 2.5 mM of acrylamide maintained the cells more viable, improved barriers abnormality and up-regulated the expression of junctional proteins of occluding, ZO-1 and claudin-1, thereby supporting the tight junctions.

### Myricitrin

Myricitrin (3´, 4´, 5´, 5, 7-five hydroxyflavone-3-O-α-L-rhamnoside) is a flavonoid naturally existing in the bark of bayberry (*Myrica cerifera*) and other plants (31, 49). Chen et al. (31) proposed if the myricitrin protects intestinal cells against acrylamide toxicity. They co-administrated the cells with 2.5, 5 or 10 µg/ml myricitrin and 5 mM of acrylamide for 48 hours. In the study, myricitrin was documented to be an efficient scavenger of various free radicals. They suggested that myricitrin prevented the proliferation of the Caco-2 cells and ROS formation, which were evoked by acrylamide treatment.

### Cocoa polyphenolic extract/constituents

Cocoa-derived products are naturally existing dietary products with antioxidant activities and many biologically important properties (32, 50). In a study done by Rodriguez-Ramiro et al. (32), they assessed the potential protective role of cocoa polyphenolic extract (containing 383.5 mg epicatechin, 116 mg catechin, 254.5 mg procyanidin and non-flavonoid components) and its main constituent. In the study, acrylamide cytotoxicity was reversed by cocoa polyphenolic extract or its polyphenols, EC and procyanidin B2, which was indicated by the inhibition of GSH consumption and ROS production, increases in the amount of gamma-glutamylcysteine synthase and glutathione-S-transferase and decreases in the caspase 3 activity. Moreover, the extract and procyanidin B2 prevented acrylamide- induced p-JNK (c-Jun N-terminal kinase) increase.

### Hydroxytyrosol

In another study done by Rodriguez-Ramiro et al. (33), the preventive effect of hydroxytyrosol against acrylamide toxicity was studied on Caco-2 cells. Hydroxytyrosol is a dietary substance that is naturally and abundantly found in olive oil, having the ability to boost antioxidant capacity of the cell, hence hampering the oxidative stress. First, hydroxytyrosol was pretreated with the doses of 5, 10, 20 and 40 µM for 20 hours. Followingly, the cells were treated with 5 mM of acrylamide. Finally, the authors suggested that ROS was prominently neutralized, caspase 3 activity was moderately decreased and increased GSH-Px and glutathione reductase activities were lowered to a basal level. Besides, the increase in p-JNK level was partially reversed. However, the depletion of GSH stores could not be hampered by hydroxytyrosol (33).

### Retinal cells

Retinal pigment epithelium cells are the outermost layer of the retina with functions of absorbing dispersed light, constituting the barrier of blood-retina and transporting of nutrients from the vascular choroid. ARPE-19 the cells of retinal pigment epithelium were obtained from a 19-year-old male donor. These cells have a highly epithelium-like appearance and proliferate fast (51).

### Carnosic acid

Carnosic acid is a plant-derived phenolic compound and found in rosemary and sage, with various biological activities, including antioxidant, antibacterial, anticarcinogenic, anti-inflammatory activities. Albalawi et al. (22) examined the possible protective impact of carnosic acid on ARPE-19 human retina cells following acrylamide treatment. First, the cells were pretreated with 10 µM of carnosic acid for 24 hours. Thereafter, 0.7 or 1 mM of acrylamide was applied to the cells for 24 hours. Carnosic acid pretreatment increased cell viability and antioxidant levels and decreased cell death and oxidant levels after acrylamide treatment. Besides, carnosic acid enhanced the decreased Nrf2 expression after acrylamide treatment.

### Mammary gland cells

Human MDA-MB-231 are epithelial mammary adenocarcinoma cells obtained from pleural effusion of a 51-year old female patient with breast cancer (52).

### Cyanidin-3-glucoside

Cyanidin-3-glucoside (Cy-3-glu) is the most widespread anthocyanin pigment type in various fruits, mainly the berries (53). Song et al. (24) employed Cy-3-glu to counter the acrylamide damage in human MDA-MB-231cells. The cells were administered with 10, 25, 50, 100 µM of Cy-3-glu for 4 hours. Then, 5 mM of acrylamide was introduced to the cells for 20 hours. The pretreatment of Cy-3-glu presented a protective role against acrylamide toxicity by reducing ROS production, recovering GSH depletion and enhancing the expression of cytoprotective enzymes. Besides, the high concentrations of Cy-3-glu (50 and 100 µM) were demonstrated to inhibit the expression of cytochrome P450 2E1.

### MAPK pathway

MAPK pathway is a very crucial pathway for modulating vital cellular events, including cellular growth, differentiation and death. It encompasses three families in mammals: c-Jun N-terminal kinase (JNK), extracellular-signal-regulated kinase (ERK) and stress-activated protein kinase (p38/SAPK) families (54). The MAPK pathway proteins are activated by serial phosphorylation events. Therefore, in the studies, the ratio of phosphorylated MAPK to non-phosphorylated MAPK is reported. In this respect, in a study done by Hong et al. (16), while pJNK/JNK and p-p38/p38 ratios increased, p-ERK/ERK ratio decreased. On the other hand, allicin pre-treatment activated ERK pathway and suppressed JNK and p38 pathways. In a study performed by Song et al. (26), acrylamide elevated the expression of p-ERK, p-p38 and p-JNK. Pretreatment with 50 µM lipoic acid for 1 hour lowered the expression of p-ERK, p-p38 and p-JNK. In a study done by Yildizbayrak and Erkan (17), 10, 100 and 1000 µM of acrylamide induced the phosphorylation of ERK, JNK and p38 protein. Cotreatment with 2.5 µM curcumin with acrylamide suppressed this increase in all acrylamide-treated group. Rodriguez-Ramiro et al. (32) also reported an increase in p-ERK/ERK, p-JNK/ JNK and p-p38/p38 ratios after acrylamide treatment. 10 µM of EC pre-treatment could not significantly change the increase of any MAPK pathway proteins. However, pretreatments with either 10 µg/ml of cocoa polyphenolic extract or 10 µM of procyanidin B2 significantly decreased inhibited the activation of all three MAPK pathway proteins aforementioned above. In another study done by Rodriguez-Ramiro et al. (33), acrylamide-induced pJNK augmentation was diminished by hydroxytyrosol pretreatments (5, 10, 20, 40 and 100 µM).

Altogether, it can be referred from *in vitro* studies associated with acrylamide toxicity that the MAPK pathway is one of the main targets to quench acrylamide toxicity as it is prominently activated following acrylamide treatment. After acrylamide treatment, the ERK, JNK and p38 proteins, which are included in the MAPK pathway, are phosphorylated, and any protective agent to decrease these proteins is considered a candidate for preventing acrylamide toxicity.

### Nrf-2 pathway

Nrf2 pathway is the pathway whereby the expression of antioxidant-related proteins is enhanced. It chiefly composes the proteins of Nrf2 and Keap1. Under normal conditions, the Nrf2 is located in the cytoplasm bound to Keap1 protein. Upon induction, Keap1 protein is degraded and Nrf located in the cytoplasm is released and translocated into the nucleus where it finally activates antioxidant-associated genes. Nrf2 signaling pathway is very important to help the organism to cope with stress when the antioxidants are depleted and oxidant levels surge. With the activation of this pathway, additional antioxidants are produced and the cell is alarmed. Regarding the Nrf2 pathway, in a study done by Li et al. (29), 5 mM of acrylamide treatment upregulated the expression of nuclear Nrf2 and downregulated the cytoplasmic Nrf2. While 48, 96 and 192 µg/ml of silymarin pretreatment significantly increased the nuclear Nrf2 amount, 12 and 24 µg/ml of silymarin could not change it when compared to the acrylamide-treated group; however, all used silymarin doses decreased cytoplasmic Nrf2 levels. As for the total Nrf levels, only 96 and 192 µg/ml of silymarin were significantly different from acrylamide-treated group. In a study done nyn Albalawi et al. (22), 0.7 or 1 mM acrylamide resulted in decreased amount of total Nrf2 at both mRNA and protein levels in the ARPE-19 cells. 10 µM of carnosic acid pre-treatment folded mRNA and protein levels of Nrf2 more than twice that of acrylamide-treated groups. Pan et al. (19) treated PC12 cells with 0.6, 1.25, 2.5 and 5 mM of acrylamide for 24 hours and detected a dose-dependent increase in nuclear Nrf2 and decrease in cytoplasmic Nrf2. They also reported an increase in the antioxidant enzymes of HO-1 and NQO-1 that are regulated by Nrf2. Then, they co-treated the cells with 0.6 mM NAC and 2.5 mM acrylamide for 24 hours. They detected an even higher increase in the co-treated group when compared to the acrylamide-treated group. Moreover, intriguingly, in the same study, when ERK 1/2, JNK and p38 inhibitors were used, Nrf2 expression was suppressed, indicating an interplay between Nrf2 and MAPK pathways.

Nrf2 pathway is a pathway utilized by acrylamide. Nevertheless, data about whether the Nrf2 increases or decreases after acrylamide application is controversial. In the light of the majority of studies, Nrf2 pathway is activated upon acrylamide and protective agents add on this increase by re-increasing Nrf2 used in order to boost antioxidant levels.

### Overall evaluation 

Briefly, all agents used against acrylamide were reported to exhibit protective activity. 5 mM concentration of acrylamide and 24-hour treatment were the most employed dose and duration in the literature. Usually, the beneficial agents were applied to the cells by pre-treatment, and acrylamide was applied after the beneficial agent separately. The protective agents were, in most studies, applied to the cells either for 24 or 48 hours. The shortest treatment of a protective agent was 30 min [hydroxytyrosol (35) and nano-cerium pretreatments (18)]. PC12 cells were the most utilized cell line, and the MAPK and Nrf2 pathways were the most studied pathways. For the pure substances, the lowest concentration of utilized protective agents was 2.5 µM belonging to curcumin (17). The highest concentration of utilized protective agents was 600 µM (0.6 mM) belonging to NAC (19). The extracts of *Ganoderma atrum*, blueberry anthocyanin, silymarin and cocoa polyphenol were the utilized extracts against acrylamide toxicity. They were used at concentrations ranging between 5 and 192 μg/ml.

According to cell culture studies, consistent with* in vivo* studies, acrylamide induces oxidative stress by leading to an increase in ROS and MDA and depletes antioxidant stores of the cells, which was indicated by decreases in GSH, SOD and CAT. Moreover, it causes DNA damage (shown by the increase in 8-OHdG, micronucleus frequency and DNA tail length) and triggers apoptosis, which was shown in the studies by the increases in Bax, caspase 3, cytochrome c, mitochondrial membrane potential and Annexin V and the decrease in Bcl2. There are also few studies, claiming that acrylamide increases the levels of inflammatory markers, TNF-α and IL-1β. All above-mentioned studies investigated whether the used protective agents were effective against acrylamide-indued toxicity, and almost all the studies pointed out that the alleged protective agent was effective against acrylamide-toxicity to some extent.

## Conclusion

Serious measures are needed to be taken prevent or alleviate the toxicity of acrylamide, to which we are daily exposed by processed foods. Utilization of a protective agent that was reviewed in the present paper with terms of *in vitro* studies is one of the ways to be protected against acrylamide toxicity. More advanced studies focusing on mitigating acrylamide toxicity should be carried out in future.
